# Modeling Chloride Transport in Concrete Under the Coupling of Extreme Saltwater Intrusion and Short-Term Dry–Wet Alternation

**DOI:** 10.3390/ma19173636

**Published:** 2026-08-27

**Authors:** Jian Pan, Xiangyu Xu, Yang Li, Siyuan Huang, Yujian Quan, Linjian Wu

**Affiliations:** 1 National Engineering Research Center for Inland Waterway Regulation, School of River and Ocean Engineering, Chongqing Jiaotong University, 66 Xuefu Road, Nan’an District, Chongqing 400074, China; panjian203336@163.com (J.P.); wljabgf@126.com (L.W.); 2 Pinglu Canal Group Co., Ltd., No. 1618, 16th Floor, Building B, Jiaotou Mansion, No. 5 Kaixuan Road, Liangqing District, Nanning 530200, China; 3 Guangxi Laboratory of Modern Canal, Pinglu Canal Group Co., Ltd., Nanning 530200, China17323014756@163.com (Y.L.);; 4 Guangxi Pinglu Canal Digital Intellgence Science and Technology Co., Ltd., No. 5 Kaixuan Road, Liangqing District, Nanning 530200, China

**Keywords:** marine concrete structure, saltwater intrusion, chloride transport model, drying–wetting ratio, short-term dry–wet alternation

## Abstract

Estuarine ship-lock concrete is exposed to chloride concentrations approaching 3.5% and operation-induced drying–wetting cycles as short as 80 min, conditions that are not adequately represented by conventional marine tidal models. This study experimentally investigated chloride ingress under six drying–wetting ratios (*D*:*W* = 0:1, 1:9, 3:7, 1:1, 7:3, and 9:1) and compared an 80 min cycle with a conventional 24 h tidal cycle. A modified Fick-based empirical model incorporating exposure time and relative elevation *E_r_*, as a proxy for the drying–wetting ratio, was developed. High-frequency cycling produced a surface convection zone approximately 2 mm deep, and the peak chloride concentration was approximately 1.3 times that under the 24 h cycle. Both the surface chloride concentration and apparent diffusion coefficient varied nonmonotonically with the drying–wetting ratio and reached their global maxima at *D*:*W* = 7:3 (*E_r_* = 0.7). Validation against the held-out 100 d measurements showed that most prediction errors were within ±25%. These results quantify chloride transport under extreme saltwater intrusion and rapid operation-controlled cycling and provide a basis for durability assessment and service-life prediction of estuarine ship-lock concrete structures.

## 1. Introduction

Chloride-induced corrosion of reinforcing steel is the leading cause of durability deterioration in reinforced concrete structures [1,2]. Against the backdrop of global climate change and the rapid expansion of inland waterway infrastructure, the durability of concrete structures exposed to tidal estuarine environments has become an increasingly pressing concern. As an important component of the New International Land–Sea Trade Corridor in western China [3], the Qingnian Ship Lock of the Pinglu Canal is located approximately 26 km upstream of the Qinzhou estuary and is exposed to an extreme saltwater-intrusion threat driven by the upstream propagation of saline tides. In addition, frequent filling and emptying operations in the lock chamber impose highly nonstationary dry–wet alternation with extremely short periods, for example approximately 80 min, on the concrete structures over long service durations. The coupled action of high-concentration chloride salts and short-term wetting–drying alternation creates an extreme corrosive environment that differs fundamentally from conventional marine tidal zones, and this environment markedly accelerates nonequilibrium moisture migration and nonlinear chloride enrichment within concrete.

Extensive research has been conducted worldwide on the durability of concrete exposed to chloride-bearing environments, with most studies focusing on atmospheric, splash, tidal, and submerged zones in marine settings [2,4,5,6]. With respect to the spatiotemporal evolution of external environmental factors, Xu et al. [5] performed long-term field exposure measurements in tidal and splash zones, where wetting–drying alternation is most pronounced, and characterized the spatiotemporal evolution of chloride ions as well as the associated surface enrichment under cyclic wetting and drying. Zhang et al. [4] further tracked the moisture distribution in unsaturated concrete along different elevations and revealed how variations in ambient humidity regulate internal matric suction and moisture gradients. Regarding the influence of internal material structure, Song et al. [2] quantified the effects of silica-fume dosage and mixture proportions on improving the pore permeability coefficient of concrete. Wang et al. [6], from the perspective of mesoscale heterogeneity, clarified how nonuniform features such as random aggregate distribution obstruct and stochastically redirect long-term chloride diffusion pathways in tidal-zone concrete. For multifactor-coupled numerical simulation and service-life prediction, Li Chunqiu et al. [7], based on the mechanics of unsaturated porous media, systematically derived the transient coupled transport mechanism of moisture and chloride ions under alternating wet and dry states. Guan et al. [8] further incorporated the superposed effects of external climatic parameters, including temperature, humidity, and wind speed, together with internal time-dependent factors such as concrete age and chloride binding, reproduced chloride migration under complex wetting–drying environments and established a comprehensive durability-based service-life calculation model for reinforced concrete. These studies generally indicate that regions subjected to dry–wet alternation, such as tidal and splash zones, experience the most severe chloride attack and that wetting–drying alternation can substantially accelerate chloride ingress [9].

Although existing Fick-based and coupled transport models have substantially advanced chloride durability assessment, their application to estuarine ship-lock concrete remains constrained by several assumptions. These models generally treat concrete as a homogeneous continuum and represent external chloride action using a constant or slowly varying surface concentration and a time-dependent apparent diffusion coefficient calibrated under immersion or conventional tidal exposure [7,8]. Such formulations emphasize diffusion and material aging but do not explicitly capture the rapid alternation among capillary absorption, evaporation-driven moisture loss, transient convection, and surface chloride crystallization or enrichment during an 80 min operating cycle. In addition, the drying–wetting effect is commonly introduced as a prescribed temporal boundary or a single empirical correction factor; therefore, the elevation-dependent drying–wetting ratio and the potentially nonmonotonic responses of surface chloride concentration and apparent diffusion coefficient cannot be adequately represented. Directly applying such models to estuarine ship locks may consequently bias the predicted chloride profiles and service-life assessment.

However, most existing studies on wetting–drying alternation are based on natural marine tidal environments, where the cycle period is primarily controlled by diurnal tides of approximately 24 h or semidiurnal tides of approximately 12 h [10]. By contrast, the wetting–drying alternation experienced by estuarine infrastructure such as the Qingnian Ship Lock is not governed by natural tides but is directly controlled by the frequent filling and emptying operations of the lock. During busy navigation seasons, rapid and repeated fluctuations in the lock water level expose the lock-wall concrete to extremely short-term dry–wet alternation, with a typical cycle of only approximately 80 min. This short-term drying–wetting environment is fundamentally different from the conventional low-frequency marine tidal environment. Under high-frequency cycling, surface evaporation and capillary water absorption in concrete do not attain the dynamic equilibrium commonly assumed for long-term cycles; consequently, chloride accumulation and depth-distribution patterns necessarily exhibit a distinct frequency effect [11]. To date, few studies have documented the effects of short-term dry–wet alternations inherent to tidal river reaches on the transport mechanisms of chlorides in concrete. The engineering community also lacks supporting field-measured data, and durability prediction models developed for long-term exposure are therefore unable to accurately evaluate the actual service life of ship-lock structures operating under high-frequency service conditions.

Accordingly, the novelty of this study lies not only in the use of an operation-controlled 80 min cycle at 3.5% NaCl, but also in how the dry–wet effect is represented in the transport model. Existing Fick-based models for wet–dry exposure commonly adopt boundary conditions calibrated under immersion or conventional 12 h and 24 h tides and describe cyclic exposure using a single temporal boundary or empirical correction factor. In contrast, the present model converts the actual ship-lock water-level history into six elevation-dependent dry–wet ratios and separately expresses surface chloride concentration *C_s_*(*t*, *E_r_*) and apparent diffusion coefficient *D_a_*(*t*, *E_r_*). By introducing the relative-elevation influence coefficient *N*(*E_r_*) into the time-dependent Fick framework, the model captures the nonmonotonic response of chloride transport to elevation and identifies the critical condition at *D*:*W* = 7:3 (*E_r_* = 0.7), which cannot be represented by a monotonic or single-factor correction. This formulation links ship-lock operating conditions directly to depth- and time-dependent chloride predictions and can support elevation-based durability zoning, targeted inspection, and maintenance planning for estuarine ship-lock concrete. The specific objectives are to

(1) Quantify the effects of the dry–wet ratio, represented by relative elevation, on chloride concentration profiles, surface chloride concentration *C_s_*, and apparent chloride diffusion coefficient *D_a_* under 3.5% NaCl exposure and an 80 min cycle;
(2) Determine the cycle-period effect by comparing chloride transport under the operation-controlled *T* = 80 min regime and the conventional marine tidal *T* = 24 h regime at *D*:*W* = 1:1;
(3) Develop and validate a Fick-based chloride transport model that incorporates exposure time t and relative elevation Er through the drying–wetting regime influence coefficient *N*(*E_r_*) for concrete exposed to extreme saltwater intrusion and short-cycle drying–wetting alternation.

## 2. Experimental Method

### 2.1. Environmental Simulation Settings

Numerical simulations conducted by our research team for seawater intrusion downstream of a typical estuarine ship lock in China show that, under normal hydrological conditions, upstream runoff dilution usually keeps the chloride concentration in the lock chamber and downstream approach channel at a relatively low level. During dry seasons or astronomical spring tides, however, high-salinity seawater from the open sea propagates landward with the tidal wave, and the salinity front can readily pass the lock location. As a result, the salinity of the downstream lock water body may increase sharply and, under extreme conditions, may approach or even reach the level of open seawater, corresponding to an NaCl concentration of approximately 3.5%. Therefore, this study used an environmental chloride concentration of 3.5% to represent the standard seawater salinity associated with extreme saltwater intrusion.

Based on operational parameters from a representative estuarine ship lock in China, a complete water-level fluctuation cycle spans roughly 80 min, which accounts for the 15 min vessel entry/exit and a 10 min chamber filling or emptying stage. The dry–wet ratio (*D*:*W*) adopted in the laboratory simulation was determined from actual operational data for one operating cycle of a typical estuarine ship lock in China. Within this 80 min cycle, the water level varies across a relative elevation of 0–10 m. The corresponding water level–time relationship dictates the drying and immersion intervals for concrete at varying heights, thus establishing the experimental dry–wet ratios. Specifically, at a 1 m relative elevation, the concrete undergoes 8 min of exposure to air and 72 min of submersion, yielding a *D*:*W* of 1:9. To simulate this short-term environment, six *D*:*W* ratios reflecting different elevations were selected: 0:1, 1:9, 3:7, 1:1, 7:3, and 9:1. Furthermore, a baseline marine tidal regime with a 24 h cycle (*T* = 24 h) [12] and a 1:1 *D*:*W* ratio was introduced to differentiate the impacts of cyclic periods on chloride penetration.

**Figure 1:**
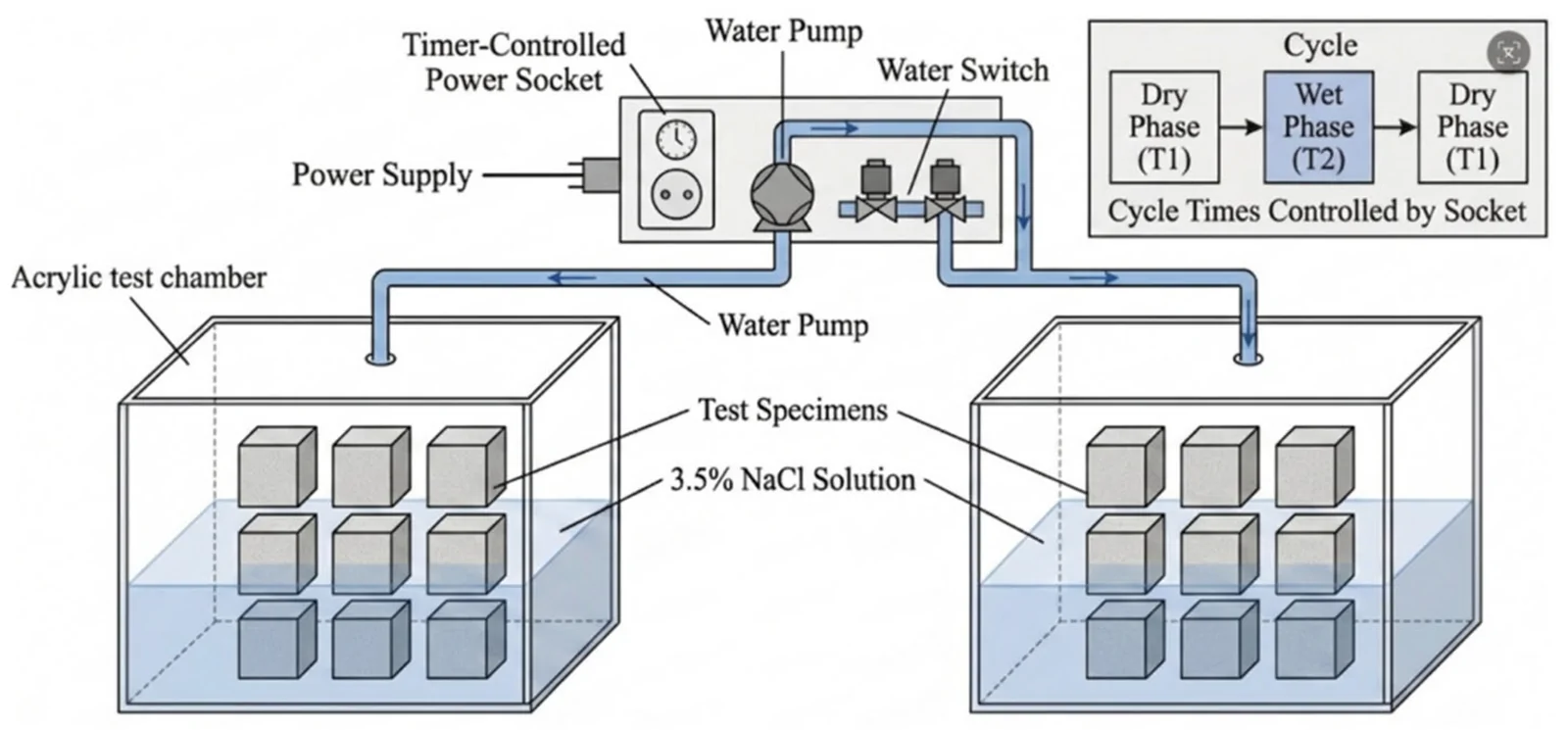
Drying–wetting cycling simulation device.

To replicate the prescribed exposure regimes, an in-house-developed cyclic drying–wetting apparatus was utilized. As illustrated in Figure 1, the setup primarily integrates bilateral testing chambers, a temporal controller, and a hydraulic pump. During simulation, fluid inflow and drainage were regulated via the automated pump driven by the time-control unit, thereby facilitating programmable variable-frequency environmental cycling. To mitigate the effects of solvent evaporation on saline concentration and ensure experimental accuracy, the exposure solution within each compartment was refreshed at 10 day intervals. Throughout the testing phase, the ambient temperature and relative humidity were strictly sustained at 20–27 °C and 70–80%, respectively.

### 2.2. Raw Materials and Specimen Preparation

Given that mass concrete is employed in the actual ship lock, all laboratory specimens were fabricated using a single batch of P.O 42.5 ordinary Portland cement (density: 3.1 × 10^3^ kg/m^3^) to mimic field conditions. The aggregate skeleton comprised continuously graded natural crushed stone (5–40 mm particle size, apparent density of 2.69 × 10^3^ kg/m^3^) and natural river sand characterized by a fineness modulus of 2.61 and an apparent density of 2.61 × 10^3^ kg/m^3^. Ordinary tap water served as the mixing agent. To optimize the binder matrix, Class F Grade I fly ash (10.1% fineness) and S95 ground granulated blast-furnace slag (specific surface area: 436 m^2^/kg) were incorporated as mineral admixtures, alongside a composite high-efficiency water-reducing agent featuring a 10–18% water reduction capability. Guided by the design codes for hydraulic mass concrete [13] and the operational demands of the lock structure, the finalized mix proportions are compiled in Table 1.

**Figure 2:**
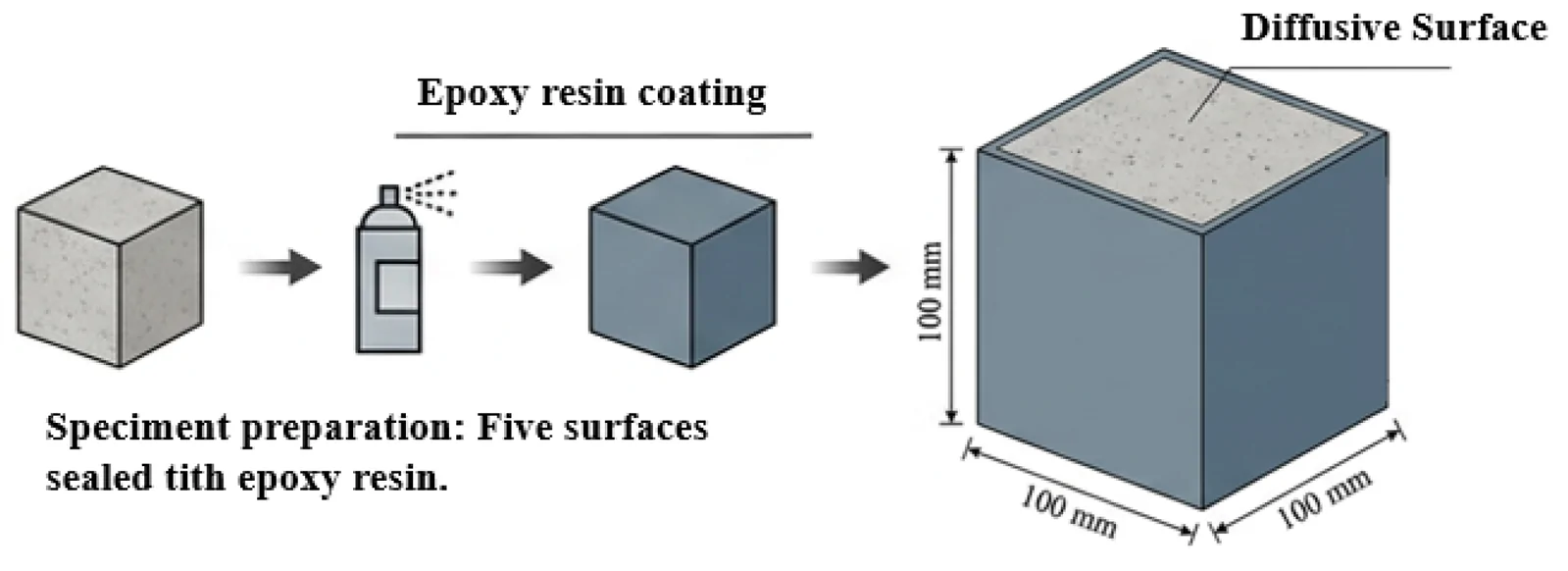
Schematic of specimen pretreatment for one-dimensional chloride transport testing.

All raw materials were weighed according to the mix proportion in Table 1 and mechanically mixed. The fresh concrete was then cast into 100 × 100 × 100 mm^3^ plastic molds. After sufficient vibration compaction and surface finishing, the molds were placed in a standard curing chamber for 24 h. The specimens were then demolded and immersed in saturated Ca(OH)_2_ solution for 28 d. Post-curing, the specimen surfaces were ground to strip off laitance, cleansed using distilled water, swabbed with ethanol, and naturally dried in a ventilated room. In order to enforce unidirectional chloride penetration, epoxy resin was applied to coat the five non-diffusive boundaries of each cube, followed by air-drying, as illustrated in Figure 2.

Seven exposure regimes were established at an environmental NaCl concentration of 3.5%: six operation-controlled regimes with *T* = 80 min and *D*:*W* = 0:1, 1:9, 3:7, 1:1, 7:3, and 9:1, together with one conventional marine tidal reference regime with *T* = 24 h and *D*:*W* = 1:1. For each of the five exposure durations (*t* = 30, 60, 100, 150, and 200 d), two cube specimens were assigned to every exposure regime. Because layerwise grinding for chloride profiling was destructive, separate specimens were used at each exposure duration. Accordingly, each exposure regime comprised 10 specimens (5 durations × 2 specimens), giving 70 specimens in total (7 regimes × 5 durations × 2 specimens). The internal and external influencing factors, including the environmental chloride concentration, dry–wet ratio, and drying–wetting period, are summarized in Table 2.

### 2.3. Test Methods

At designated exposure intervals (*t* = 30, 60, 100, 150, and 200 d), the corresponding concrete specimens were harvested for testing. A specialized grinding machine facilitated the collection of powder samples, progressing layer by layer from the exposed diffusion boundary downward. To characterize the convection zone, powder was obtained at 1 mm steps within the initial 0–4 mm depth; beyond this, from 4 to 20 mm, the sampling interval was increased to 2 mm. After capturing the powder of each layer into separate, well-labeled transparent zip-lock bags, the free chloride content (namely water-soluble chloride concentration) was measured in accordance with ASTM C1218/C1218M-20 [15]. According to the precision statement in this standard, the single-laboratory standard deviation is 0.0013% chloride by mass of mortar or concrete, and two tests conducted in the same laboratory on the same material are not expected to differ by more than 0.0037%. These values describe the uncertainty of the chemical determination itself; they do not include variability arising from specimen heterogeneity or layerwise sampling.

## 3. Test Results and Analysis

### 3.1. Chloride Concentration Profiles

**Figure 3:**
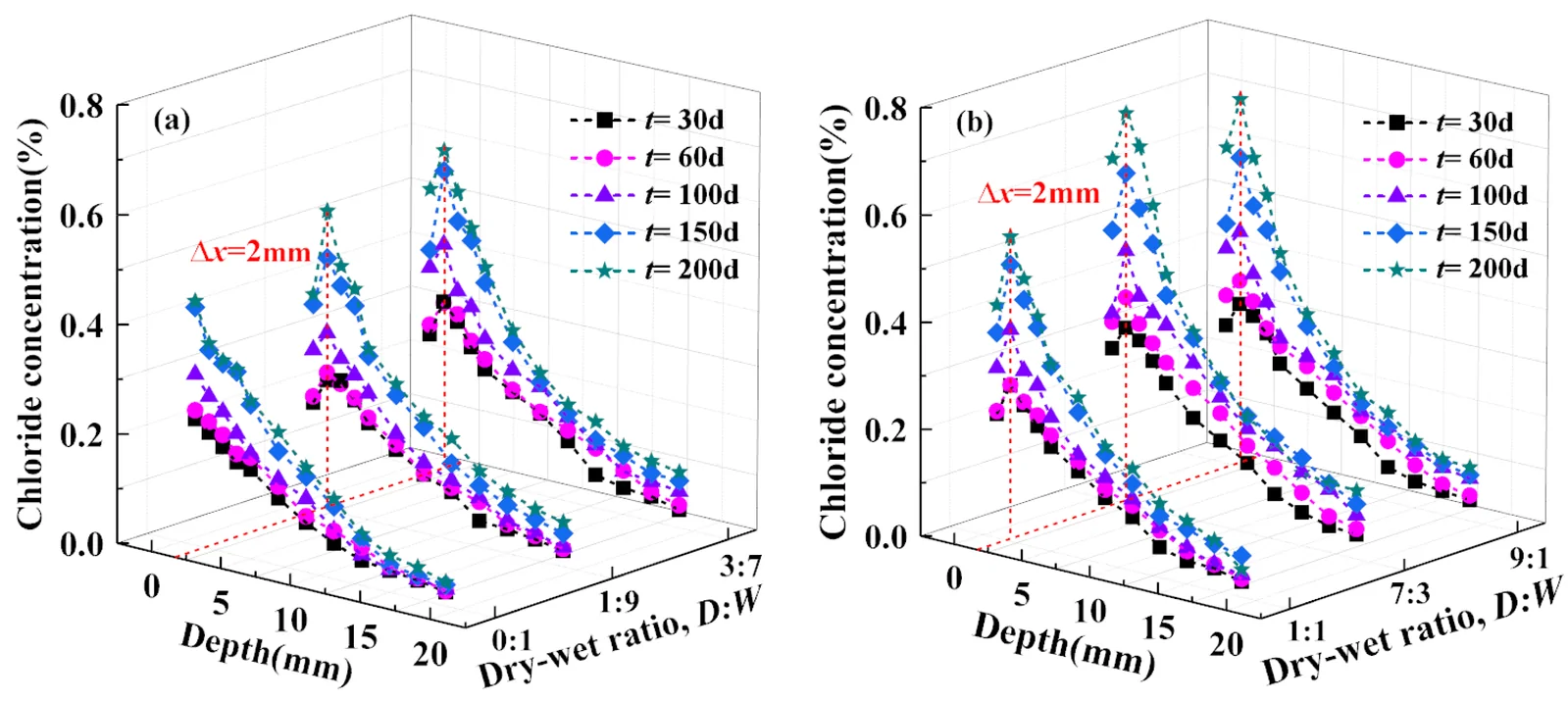
Chloride concentration profiles with *T* = 80 min: (**a**) *D*:*W* = 0:1, 1:9, 3:7; (**b**) *D*:*W* = 1:1, 7:3, 9:1.

Figure 3 delineates how chloride concentrations vary across space and time within concrete exposed to an aggressive saltwater environment. The simulation involved a 3.5% chloride solution, a cyclic drying–wetting duration (*T* = 80 min), and six distinct dry-to-wet ratios (*D*:*W* = 0:1, 1:9, 3:7, 1:1, 7:3, and 9:1). From these graphical data, several key findings emerge:

(1) As the exposure duration increased from 30 d to 200 d, the chloride levels inside the ship lock concrete exhibited a time-dependent upward trend, reinforcing the conclusions established in prior literature [16,17].
(2) Under fully immersed conditions, the chloride concentration gradually decreased with increasing depth, and no convection zone was observed. Under all wetting–drying conditions, however, the chloride concentration curve did not reach its maximum value at the exposed surface (*x* = 0 mm). Instead, the curve exhibited a distinct peak at approximately 2 mm below the surface, as indicated by the red dashed line in the figure (Δ*x* = 2 mm). Beyond this peak, the concentration decreased sharply with increasing depth. This maximum-concentration phenomenon is a distinctive feature of chloride ingress under dry–wet alternation, in contrast to fully immersed exposure, and it has been widely confirmed in previous studies [18].
(3) The convection-zone depth (or its influence depth), denoted as Δ*x*, represents the location where the peak surface-layer chloride concentration occurs. In this study, Δ*x* was determined experimentally from the layerwise chloride profiles using an objective peak-position criterion: the first measured depth at which the free chloride concentration reached its maximum and then decreased toward the interior. Because powder samples within the 0–4 mm surface region were collected at 1 mm intervals, the depth resolution was 1 mm. The concentration maximum occurred at 2 mm for all drying–wetting regimes and exposure durations; therefore, Δ*x* = 2 mm was obtained directly from the experimental data rather than calculated from concentration gradients or adopted from previous studies. Using this peak location as a boundary, the internal chloride profile within the concrete can be separated into two distinct regions: a convection zone and a stable diffusion zone [19]. Consequently, the ensuing analysis herein concentrates exclusively on the transport behavior and distribution patterns within the stable diffusion zone, omitting the chloride variations inside the convection zone.

### 3.2. Effect of Dry–Wet Ratio on Chloride Concentration Profiles

**Figure 4:**
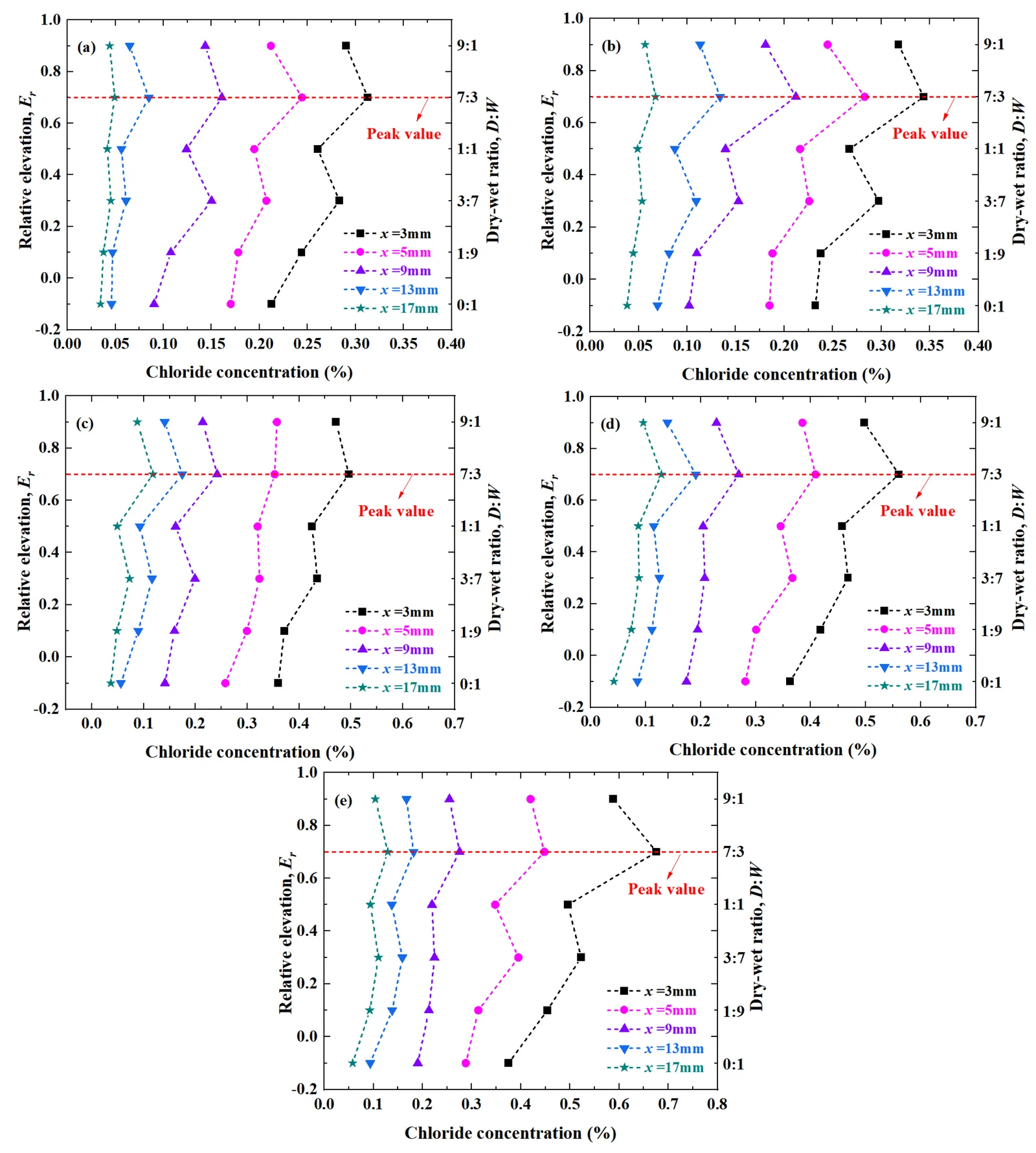
Chloride concentration profiles in concrete under different dry–wet ratios: (**a**) *t* = 30 d; (**b**) *t* = 60 d; (**c**) *t* = 100 d; (**d**) *t* = 150 d; (**e**) *t* = 200 d.

The dry–wet ratio determines the durations of capillary absorption and moisture evaporation during wetting–drying alternation and therefore significantly affects the chloride ingress depth. On the basis of the test results obtained under different drying–wetting regimes (Figure 4), the chloride concentration profiles exhibited the following characteristics:

(1) At the same exposure duration, the chloride concentration first increased, then decreased, then increased again, and finally decreased as the drying–wetting regime intensified with increasing elevation. In other words, the chloride concentration exhibited an M-shaped trend with increasing drying duration. The maximum chloride concentration in the ship lock concrete occurred at a relative elevation of 0.7, corresponding to a dry–wet ratio of 7:3. This behavior can be attributed to the fact that, as elevation increased, the saltwater immersion duration of the lock concrete decreased, whereas the pore-solution evacuation depth in the shallow concrete and the capillary negative pressure in the surface concrete increased during drying. When the concrete was subsequently rewetted, the rate at which the concrete surface absorbed saline solution increased, and the chloride penetration rate increased accordingly. This process increased the surface chloride content and further accelerated chloride ingress into the concrete.
(2) As elevation increased further, the total immersion duration of the concrete decreased, which reduced the total chloride content in the concrete and consequently lowered the surface chloride content. This result indicates that chloride-induced deterioration of concrete under different drying–wetting regimes cannot be described by a simple qualitative rule. This study demonstrates that the severity of chloride attack in the drying–wetting zone of ship lock concrete first increases, then decreases, then increases again, and finally decreases as the drying–wetting regime intensifies with increasing elevation.

### 3.3. Effect of Wetting–Drying Alternation Period on Chloride Concentration Profiles

**Figure 5:**
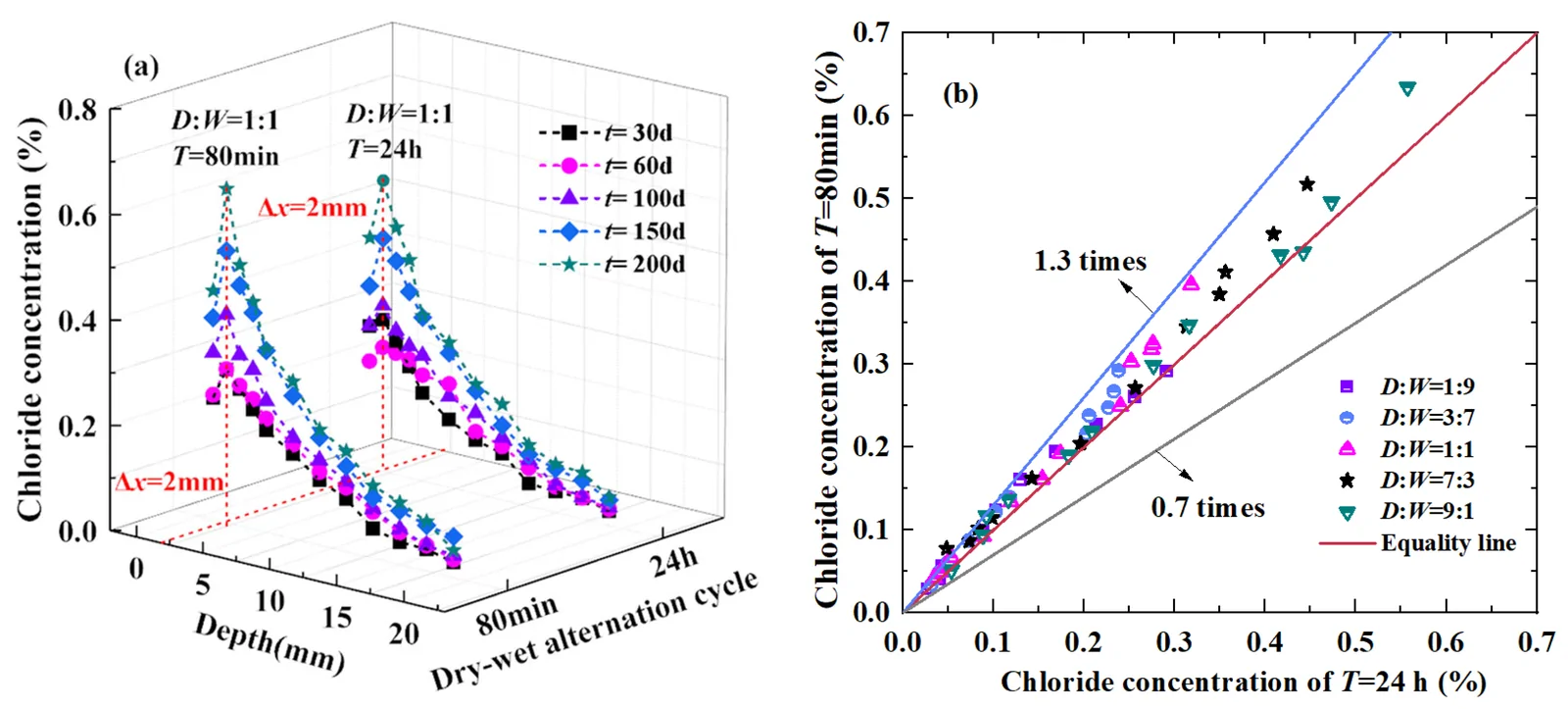
Comparison of chloride concentrations under different dry–wet alternation periods: (**a**) *T* = 80 min vs. *T* = 24 h; (**b**) quantitative comparison.

To further reveal the influence of drying–wetting period on chloride transport depth and concentration distribution, Figure 5 compares the chloride concentration data measured under the dry–wet ratio *D*:*W* = 1:1 for a short period of *T* = 80 min and a longer period of *T* = 24 h. The analysis indicates the following:

(1) Under different drying–wetting periods, as the exposure duration increased from 30 d to 200 d, the chloride concentration curves at all depths showed an evident upward shift. This result indicates that chloride ions from the external environment were continuously transported into the concrete and accumulated with increasing exposure time.
(2) At the same exposure duration, the convection-zone depths of concrete subjected to drying–wetting periods of *T* = 80 min and *T* = 24 h were essentially identical, with no significant frequency-induced shift. Because of the convection mechanism governed by surface moisture evaporation and capillary absorption, the chloride concentration under both periods displayed a typical increase-then-decrease distribution with increasing depth. The peak chloride concentration in both cases remained at a depth of 2 mm below the surface. This finding indicates that the drying–wetting period mainly affects the amount of ion accumulation but does not significantly alter the influence depth of the convection zone.
(3) When the external conditions were fixed, the chloride concentration and its peak value in concrete subjected to the drying–wetting period *T* = 80 min were generally higher than those in concrete subjected to *T* = 24 h. The quantitative analysis in Figure 5b shows that all data points lie above the 1:1 equivalence line (red line), and most points are tightly distributed within the +1.3-times band (blue control line). This distribution directly indicates that the chloride concentration and peak value under the short period *T* = 80 min were approximately 1.3 times those under the drying–wetting period *T* = 24 h. This result occurs because *T* = 80 min produces a larger number of dry–wet alternations within the same total exposure duration. The relatively short-term drying–wetting process causes more frequent pore-water evacuation and saline-solution uptake in the surface capillaries, thereby greatly enhancing convective transport driven by capillary absorption. Meanwhile, the high-frequency evaporation process intensifies moisture loss and promotes strong chloride concentration and crystallization accumulation within the convection zone. This superposed high-frequency convection–concentration effect directly increases the surface concentration peak, thereby increasing the concentration-gradient driving force for chloride transport into the stable diffusion zone and ultimately leading to a marked increase in the overall internal chloride concentration.

## 4. Discussion

### 4.1. Fick’s Second Law

To evaluate the chloride concentration profiles within the stable diffusion zone of cementitious materials, Fick’s second law is widely adopted [20]. In scenarios involving one-dimensional chloride transport, the error-function-based analytical solution is formulated as

(1) $$C\left(x,t\right)=C_{s}\cdot \left[1-\mathrm{erf}\left(\frac{x}{2\sqrt{D_{a}\cdot t}}\right)\right]$$

where *C*(*x*,*t*) is the free chloride content (%) situated at a specific depth *x* and exposure duration *t*; *C_s_* is the surface chloride concentration (%); *D_a_* is the apparent chloride diffusion coefficient (m^3^/s); and erf( ) is the Gaussian error function.

Numerous studies have shown that regression fitting can be used to characterize the time-dependent behavior of *C_s_* [21,22,23]. In this study, *C_s_*(*t*) was described by a logarithmic function as follows:

(2) $$C_{s}\left(t\right)=A\cdot \ln\left(t\right)+B$$

where *A* and *B* are fitting parameters.

Based on the power-law formulation introduced by Thomas [24], the temporal evolution of the apparent diffusion coefficient, *D_a_*, can be mathematically modeled as

(3) $$D_{a}\left(t\right)=\frac{D_{r}}{1-m}{\left(\frac{t_{r}}{t}\right)}^{m}$$

where *D_r_* is the reference chloride diffusion coefficient (m^3^/s) corresponding to a reference age of *t* = 28 d; *t_r_* is the specific reference exposure age fixed at 28 d; and *m* is the aging factor.

To summarize, the experimental chloride data gathered across various exposure durations, penetration depths, and wet–dry conditions (i.e., at distinct elevations) serve as the foundation to deduce the time-dependent surface concentration *C_s_*(*t*) and diffusion coefficient *D_a_*(*t*). By integrating these values with Equation (3), non-linear regression analysis can be executed to back-calculate critical transport parameters (*D_r_* and *m*). This workflow ultimately yields a predictive chloride ingress model for the concrete structure.

### 4.2. Effect of Dry–Wet Ratio on Chloride Transport Parameters

#### 4.2.1. Surface Chloride Concentration

**Figure 6:**
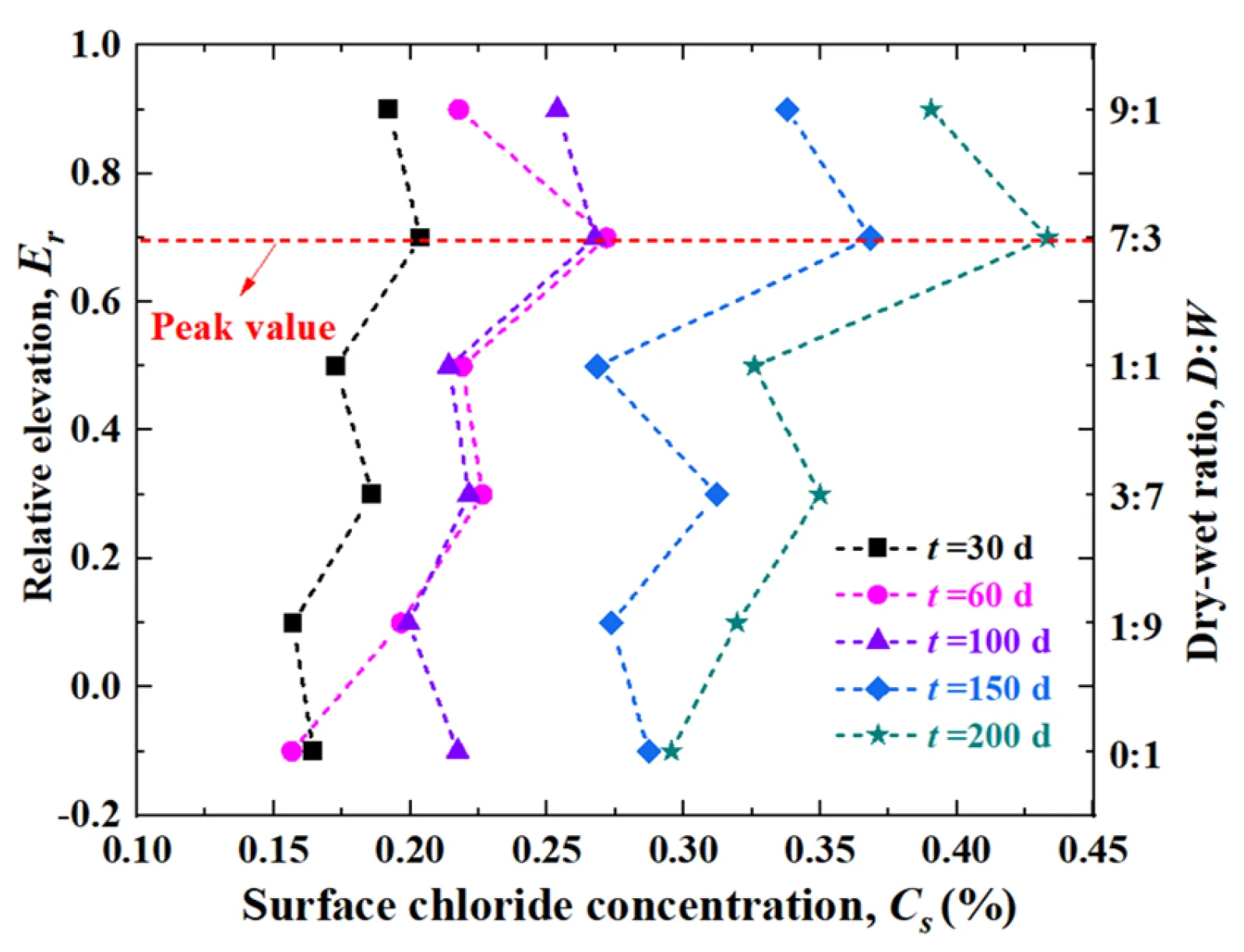
Variation in surface chloride concentration with relative elevation (dry-wet ratio).

The response of surface chloride concentration to various drying–wetting regimes under a 3.5% environmental chloride salinity is illustrated in Figure 6. Based on these graphical trends, several key phenomena can be distinguished:

(1) The dry-wet ratio, expressed as relative elevation, had a pronounced nonlinear effect on the surface chloride concentration. At the same exposure duration, as the relative elevation *E_r_* increased, corresponding to an increase in *D*:*W* from 0:1 to 9:1 on the right-hand axis, the surface chloride concentration *C_s_* did not increase monotonically. Instead, it exhibited a complex fluctuating trend, approximately resembling a sawtooth or M-shaped pattern. This result indicates that the allocation of drying and wetting durations plays a critical regulatory role in surface chloride accumulation.
(2) As explicitly indicated by the red dashed line labeled Peak value in the figure, the maximum surface chloride concentration consistently occurred at the relative elevation *E_r_* = 0.7, corresponding to *D*:*W* = 7:3. When the dry-wet ratio reached 7:3, the relatively long drying duration allowed the pore solution in the surface concrete to be substantially evacuated, generating a high capillary negative pressure. During the subsequent short wetting stage, strong capillary suction promoted rapid ingress of high-concentration saline solution. The coupled action of intense suction and long-term evaporation-induced concentration caused the chloride accumulation at this position to reach its maximum. When the dry-wet ratio further increased to 9:1, the wetting duration became too short for the concrete to absorb sufficient saline solution, and the surface chloride concentration consequently decreased.

#### 4.2.2. Apparent Chloride Diffusion Coefficient

**Figure 7:**
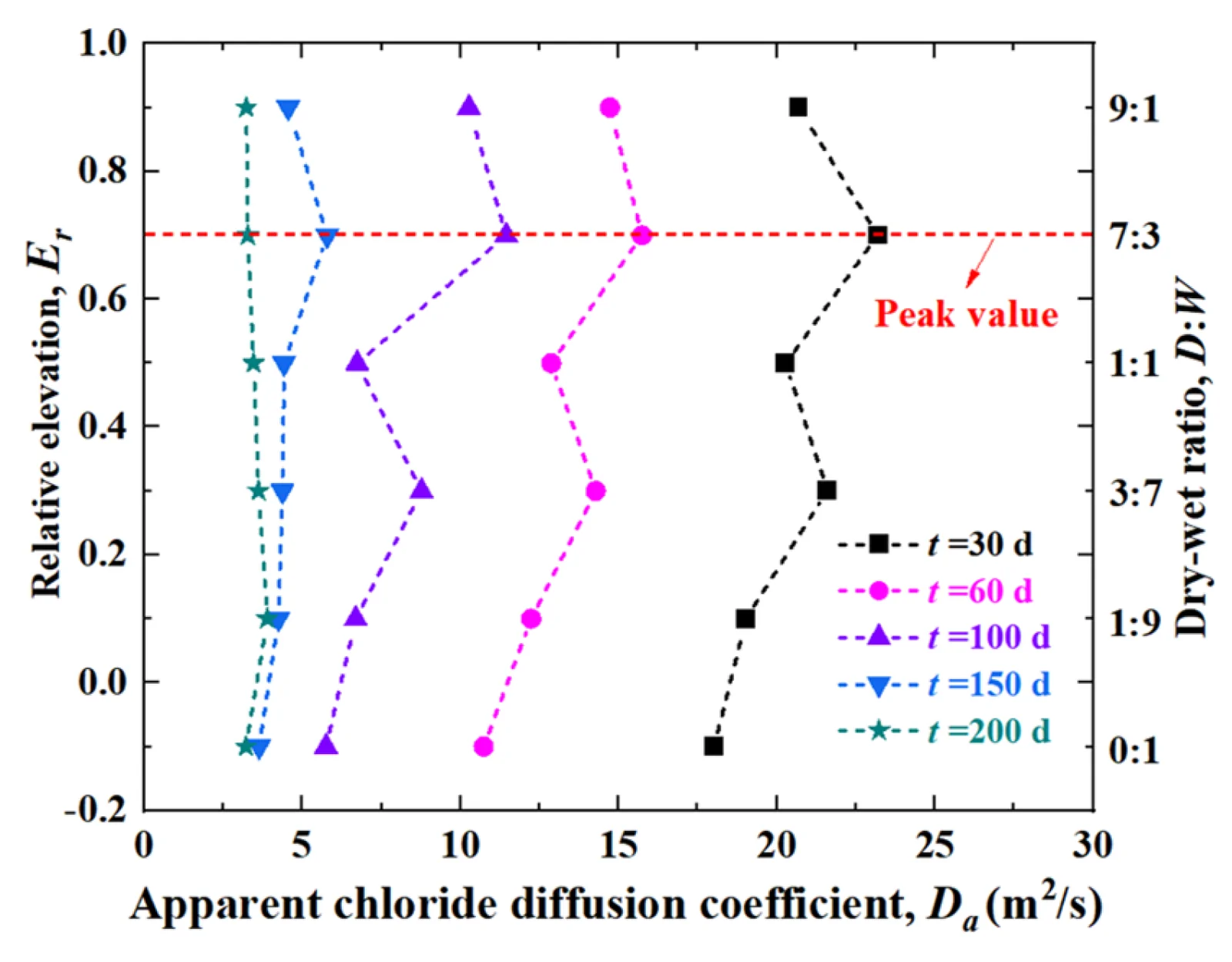
Variation in the apparent chloride diffusion coefficient with relative elevation.

Figure 7 depicts the evolution of the apparent chloride diffusion coefficient across distinct wet-dry cycles (plotted as relative elevations) at a 3.5% bulk chloride concentration. Based on the illustrated results, the following points are observed:

(1) The apparent chloride diffusion coefficient decreased significantly with increasing exposure duration. This phenomenon mainly occurs because continued hydration inside the concrete and the products formed by the binding of free chloride ions, such as Friedel’s salt, progressively fill the capillary pores as the concrete ages. The micro-pore structure therefore becomes denser, which effectively hinders further chloride diffusion into the interior.
(2) The dry–wet ratio, expressed as relative elevation, had a pronounced nonlinear effect on the apparent chloride diffusion coefficient. At the same exposure duration, as the relative elevation Er increased, corresponding to an increase in *D*:*W* from 0:1 to 9:1 on the right-hand axis, the apparent chloride diffusion coefficient *D_a_* neither decreased nor increased monotonically. Instead, it exhibited a complex fluctuating trend, approximately resembling a sawtooth or M-shaped pattern. This result indicates that the allocation of drying and wetting durations plays a critical regulatory role in the chloride transport rate within concrete. As explicitly indicated by the red dashed line (labeled “Peak value”) in the figure, the maximum apparent chloride diffusion coefficient consistently occurred at *E_r_* = 0.7, corresponding to *D*:*W* = 7:3. Regardless of exposure duration, the extremum of each curve was always located under the *D*:*W* = 7:3 condition. When the dry–wet ratio reached 7:3, prolonged drying caused strong concentration of pore solution in the surface layer, and strong capillary suction rapidly drew high-concentration saline solution into the concrete during the subsequent short wetting stage. This extreme wetting–drying process not only established a large concentration-gradient driving force at the surface but may also have aggravated drying-shrinkage microcracking in the surface pores, providing preferential channels for deeper chloride migration. Consequently, the apparent diffusion rate reached a global maximum under this condition. When the dry–wet ratio further increased to 9:1, the wetting duration became too short for the external saline solution to provide a sustained and connected moisture-transport carrier for the interior; inward diffusion pathways were therefore obstructed, and the apparent chloride diffusion coefficient decreased markedly.

## 5. Chloride Transport Model

### 5.1. Model Development

#### 5.1.1. Surface Chloride Concentration

**Figure 8:**
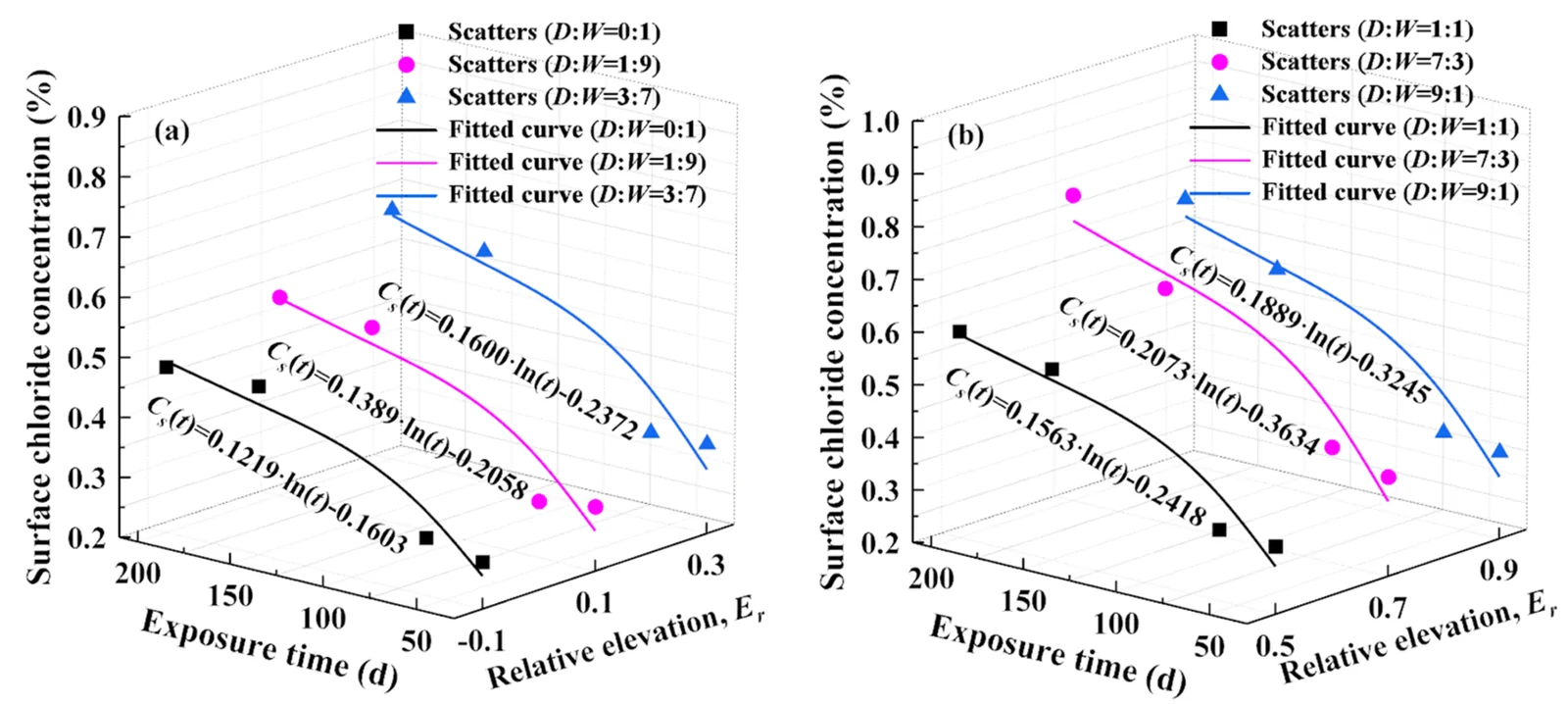
Variation and fitting of *C_s_* with t at different relative elevations: (**a**) *E_r_* = −0.1, 0.1, 0.3; (**b**) *D*:*W* = 0.5, 0.7, 0.9.

To establish a time-dependent prediction model for surface chloride concentration and verify its reliability, this study grouped the experimental data obtained at different exposure durations. Specifically, the measured chloride concentration data at *t* = 30 d, 60 d, 150 d, and 200 d were selected as the fitting dataset, and the measured data at *t* = 100 d were reserved as an independent validation dataset to examine the predictive accuracy of the theoretical model. In the modeling analysis, nonlinear least-squares regression based on Equation (1) was first applied to the measured chloride concentration profiles in concrete under each dry–wet ratio, and the corresponding discrete values of surface chloride concentration *C_s_* were inversely extracted. Subsequently, based on Equation (2), a logarithmic function was introduced to fit the variation in the extracted C*_s_* with exposure time i, as shown in Figure 8. The fitting results indicate that, under all dry–wet ratio conditions, a significant and strong logarithmic correlation existed between the surface chloride concentration *C_s_* and exposure time *t*.

After establishing that the surface chloride concentration *C_s_* increased logarithmically with exposure time *t*, satisfying the relationship *C_s_*(t) = *A*·ln(*t*) + *B* [25], this study further analyzed the mathematical relationships between the fitting parameters *A* and *B* and the relative elevation *E_r_*, namely the dry–wet ratio [26], to enable unified prediction of chloride concentration at different relative elevations.

**Figure 9:**
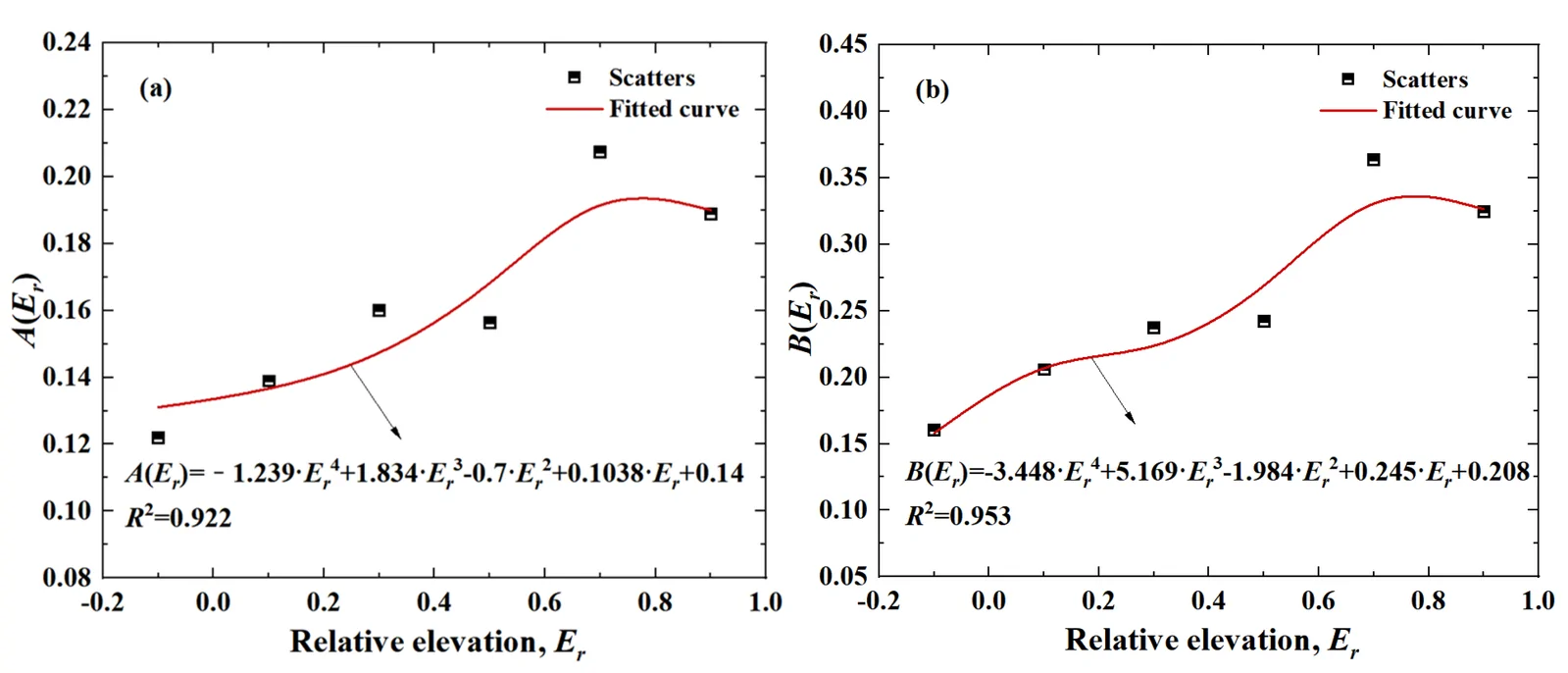
Variation and fitting of the *C_s_* model parameters *A* and *B* with relative elevation: (**a**) *A*(*E_r_*); (**b**) *B*(*E_r_*).

The fitting parameters *A* and *B* obtained under each condition were extracted and regressed against the corresponding relative elevation *E_r_* [25,27,28]. As shown in Figure 9, a fourth-order polynomial was introduced to perform nonlinear fitting of the scatter data. The results show that the fitted curves accurately captured the nonlinear fluctuations of the parameters with relative elevation, with correlation coefficients R^2^ of 0.922 and 0.953, respectively, indicating high goodness of fit. Finally, the fitted functions *A*(*E_r_*) and *B*(*E_r_*), which characterize the spatial distribution of wetting–drying alternation, were substituted into the original logarithmic equation. This procedure yielded a bivariate prediction model for surface chloride concentration that simultaneously considers exposure time *t* and relative elevation *E_r_*, namely the dry–wet ratio, under extreme chloride concentration and short-cycle wetting–drying alternation. The complete expression is given in Equation (4).

(4) $$\left\{\begin{array}{l} C_{s}\left(t\right)=A\left(E_{r}\right)\cdot \ln(t)+B\left(E_{r}\right)\\ A\left(E_{r}\right)=-1.239\cdot {E_{r}}^{4}+1.834\cdot {E_{r}}^{3}-0.7\cdot {E_{r}}^{2}+0.1038\cdot E_{r}+0.14\\ B\left(E_{r}\right)=3.448\cdot {E_{r}}^{4}-5.169\cdot {E_{r}}^{3}+1.984\cdot {E_{r}}^{2}-0.245\cdot E_{r}-0.208 \end{array}\right.$$

#### 5.1.2. Chloride Diffusion Coefficient

**Figure 10:**
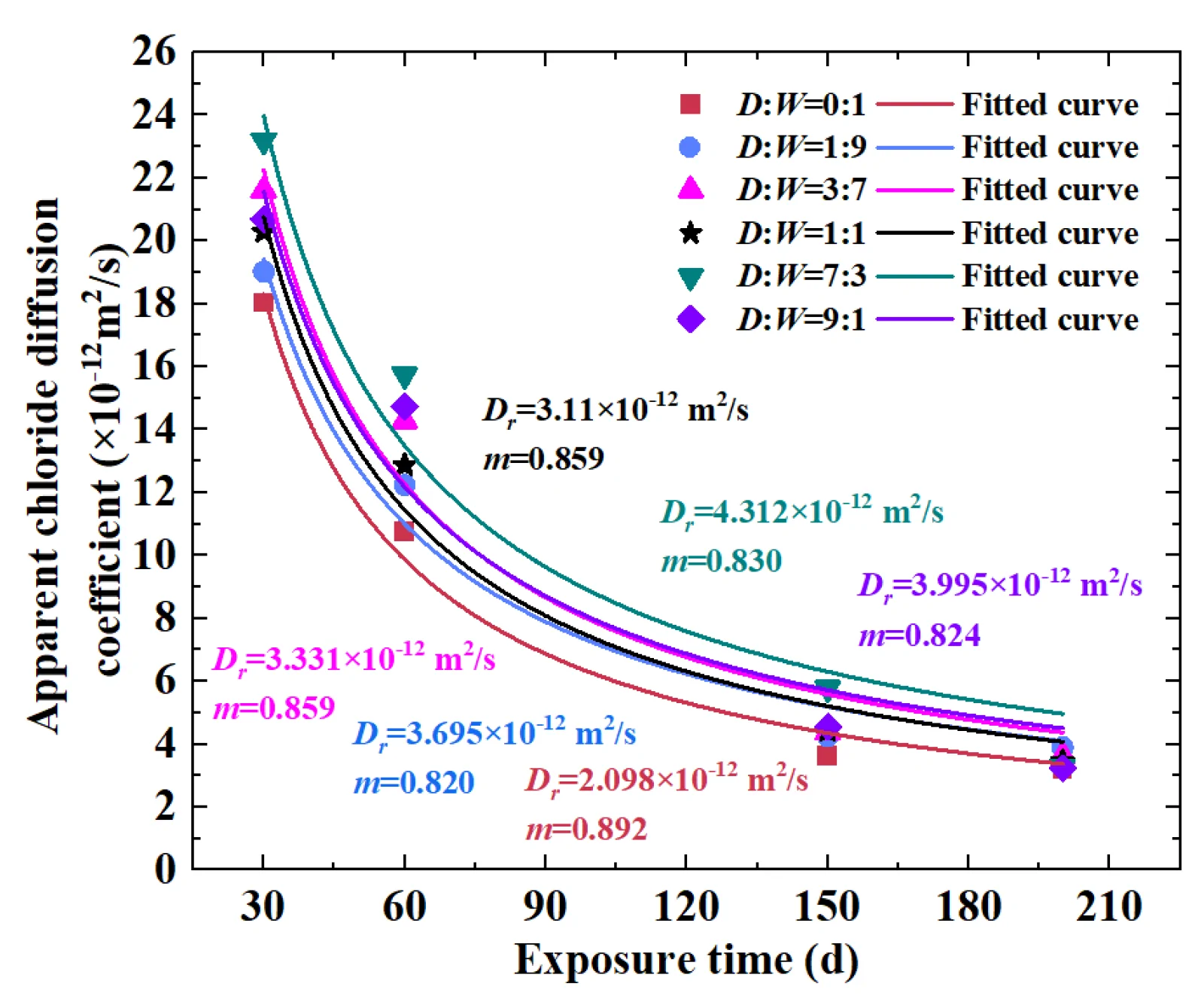
Variation in *D_a_* with *t* and characteristic parameters *D_r_* and *m*.

The empirical *D_a_* scatters for concrete specimens across diverse dry–wet ratios are summarized in Figure 10 to illustrate the variation in the apparent chloride diffusion coefficient *D_a_* over exposure time *t*. By applying the classical time-dependent attenuation model for the diffusion coefficient formatted in Equation (3), regression analysis was executed on the experimental datasets of each individual group. Consequently, two key model parameters defining the transport behavior under each drying–wetting regime—the reference chloride diffusion coefficient *D_r_* and the aging attenuation exponent *m*—were inversely extracted. The strong consensus between the fitted curves and the measured scatter data directly validates the nonlinear attenuation of *D_a_* as a function of increasing age.

To quantitatively describe the influence of different drying–wetting regimes on chloride diffusion in concrete, this study normalized the reference chloride diffusion coefficient *D_r_*. Because *D_r_* reached its peak at the relative elevation *E_r_* = 0.7, this value was selected as the reference value, and the relative-elevation influence coefficient *N*(*E_r_*) was defined accordingly, as expressed in Equation (5).

(5) $$N\left(E_{r}\right)=\frac{D_{r}\left(E_{r}\right)}{D_{r}\left(E_{r}=0.7\right)}$$

where *D_r_*(*E_r_*) is the reference chloride diffusion coefficient (m^2^/s) in the concrete specimen at a given relative elevation *E_r_*; *D_r_*(*E_r_* = 0.7) is the reference chloride diffusion coefficient (m^2^/s) in the concrete specimen when *E_r_* = 0.7; and *N*(*E_r_*) denotes the relative-elevation influence coefficient in Equation (5).

**Figure 11:**
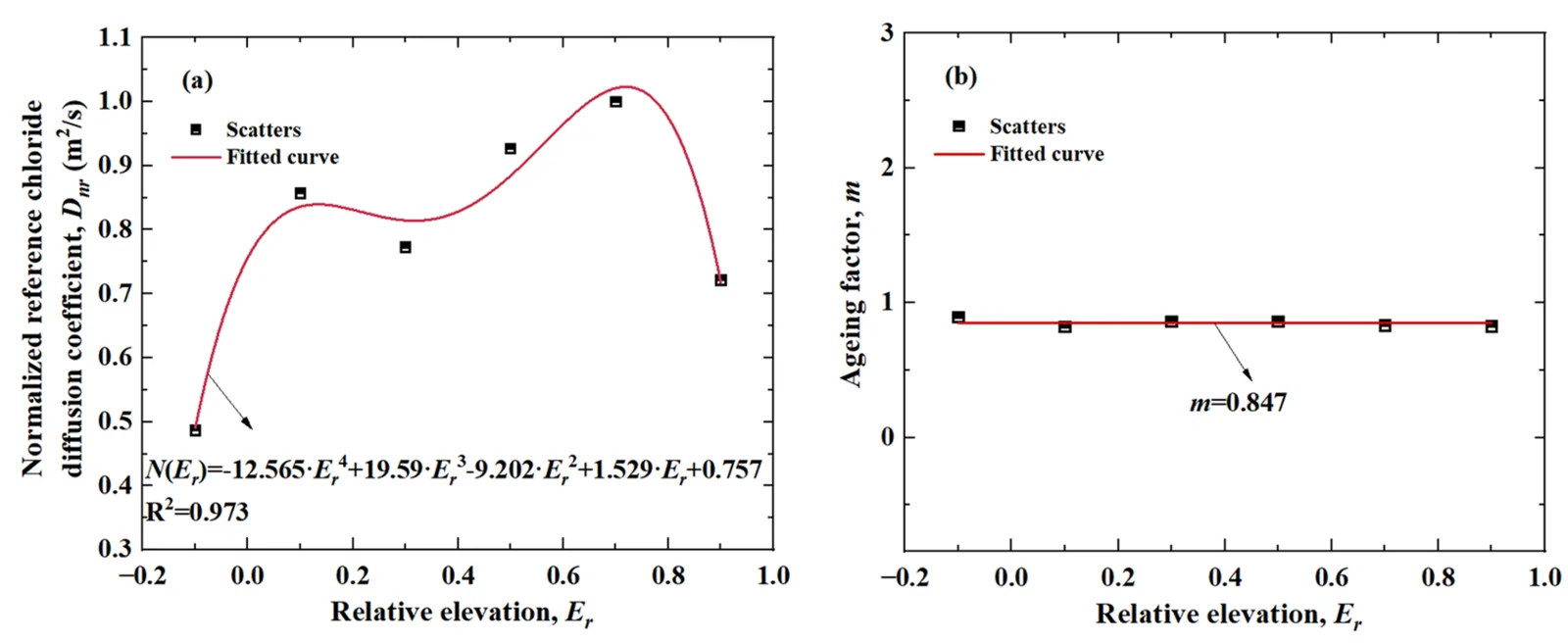
Regression analysis of the parameters: (**a**) *N*(*E_r_*); (**b**) *m*.

The discrete values of *N*(*E_r_*) under each condition were calculated using Equation (5), and nonlinear regression analysis was then performed between *N*(*E_r_*) and the relative elevation Er, as shown in Figure 11a. The analysis shows that *N*(*E_r_*) first increased and then decreased with increasing Er, that is, with increasing dry–wet ratio, and reached its peak at *E_r_* = 0.7 (*D*:*W* = 7:3). A fourth-order polynomial was introduced to fit the spatial distribution, and the fitted curve provided an excellent representation of this nonlinear trend.

The group-specific aging exponents obtained from the regressions in Figure 10 ranged from 0.820 to 0.892, with a mean of 0.847 and a coefficient of variation of approximately 3.3%. As shown in Figure 11b, these values exhibited no systematic dependence on Er within the examined drying–wetting regimes. Therefore, *m* = 0.847 was adopted as a common representative value in Equations (6) and (7) to limit the number of free parameters and avoid overfitting the four-age calibration dataset. This simplification is considered reasonable for the present specimens because the concrete composition, curing history, bulk chloride concentration, temperature, and cycle period were kept unchanged, while only the drying–wetting ratio varied. Nevertheless, m is an empirical aging parameter and may depend on binder composition, water-to-binder ratio, curing, temperature, chloride binding, and exposure regime [21,24]. Moreover, the power-law formulation treats m as constant over the calibrated time interval; thus, the adopted value should be understood as an interval-average descriptor, rather than evidence that the aging behavior is intrinsically time-invariant. Accordingly, *m* = 0.847 should be applied only to the tested material system and the 30–200 d calibration range. For other concrete mixtures, salinities, temperatures, cycle periods, or longer exposure durations, m should be recalibrated; where sufficient data are available, condition-specific m values or an explicit function *m*(*E_r_*) should be used to reduce prediction uncertainty.

By substituting the regression-derived values of *N*(*E_r_*) and *m* into Equation (3), a time-dependent model for the *D_a_* was established (Equation (6)). This model explicitly incorporates the influence of relative elevation—specifically the dry–wet ratio—under short-term wetting–drying alternations. Ultimately, it serves as an effective tool for predicting concrete diffusion coefficients across diverse environmental regimes and elevations, offering valuable theoretical backing for the durability and service-life assessment of concrete structures.

(6) $$\left\{\begin{array}{l} D_{a}(t,E_{r})=\frac{D_{r}(E_{r})}{1-m}{\left(\frac{t_{r}}{t}\right)}^{m}\\ D_{r}(E_{r})=N(E_{r})\cdot D_{r}(E_{r}=0.7)\\ N(E_{r})=-12.565\cdot {E_{r}}^{4}+19.59\cdot {E_{r}}^{3}-9.202\cdot {E_{r}}^{2}+1.529\cdot E_{r}+0.757\\ D_{r}(E_{r}=0.7)=4.312\times 10^{-12}\;m^{2}/s\\ m=0.847 \end{array}\right.$$

In summary, by combining Equations (1), (4), and (6), a chloride transport prediction model that accounts for the effect of the drying–wetting regime, corresponding to different elevations, under extreme saltwater intrusion and short-cycle wetting–drying alternation can be established. The model is expressed in Equation (7).

(7) $$\left\{\begin{array}{l} C(x,t,E_{r})=C_{s}(t,E_{r})\cdot \left[1-erf(\frac{x}{2\sqrt{D_{a}(t,E_{r})\cdot t}})\right]\\ C_{s}(t,E_{r})=A\left(E_{r}\right)\cdot \ln(t)+B\left(E_{r}\right)\\ A\left(E_{r}\right)=-1.239\cdot {E_{r}}^{4}+1.834\cdot {E_{r}}^{3}-0.7\cdot {E_{r}}^{2}+0.1038\cdot E_{r}+0.14\\ B\left(E_{r}\right)=3.448\cdot {E_{r}}^{4}-5.169\cdot {E_{r}}^{3}+1.984\cdot {E_{r}}^{2}-0.245\cdot E_{r}-0.208\\ D_{a}(t,E_{r})=\frac{D_{r}(E_{r})}{1-m}{\left(\frac{t_{r}}{t}\right)}^{m}\\ D_{r}(E_{r})=N(E_{r})\cdot D_{r}(E_{r}=0.7)\\ N(E_{r})=-12.565\cdot {E_{r}}^{4}+19.58\cdot {E_{r}}^{3}-9.202\cdot {E_{r}}^{2}+1.529\cdot E_{r}+0.757\\ D_{r}(E_{r}=0.7)=4.312\times 10^{-12}\;m^{2}/s\\ m=0.847 \end{array}\right.$$

### 5.2. Model Validation

**Figure 12:**
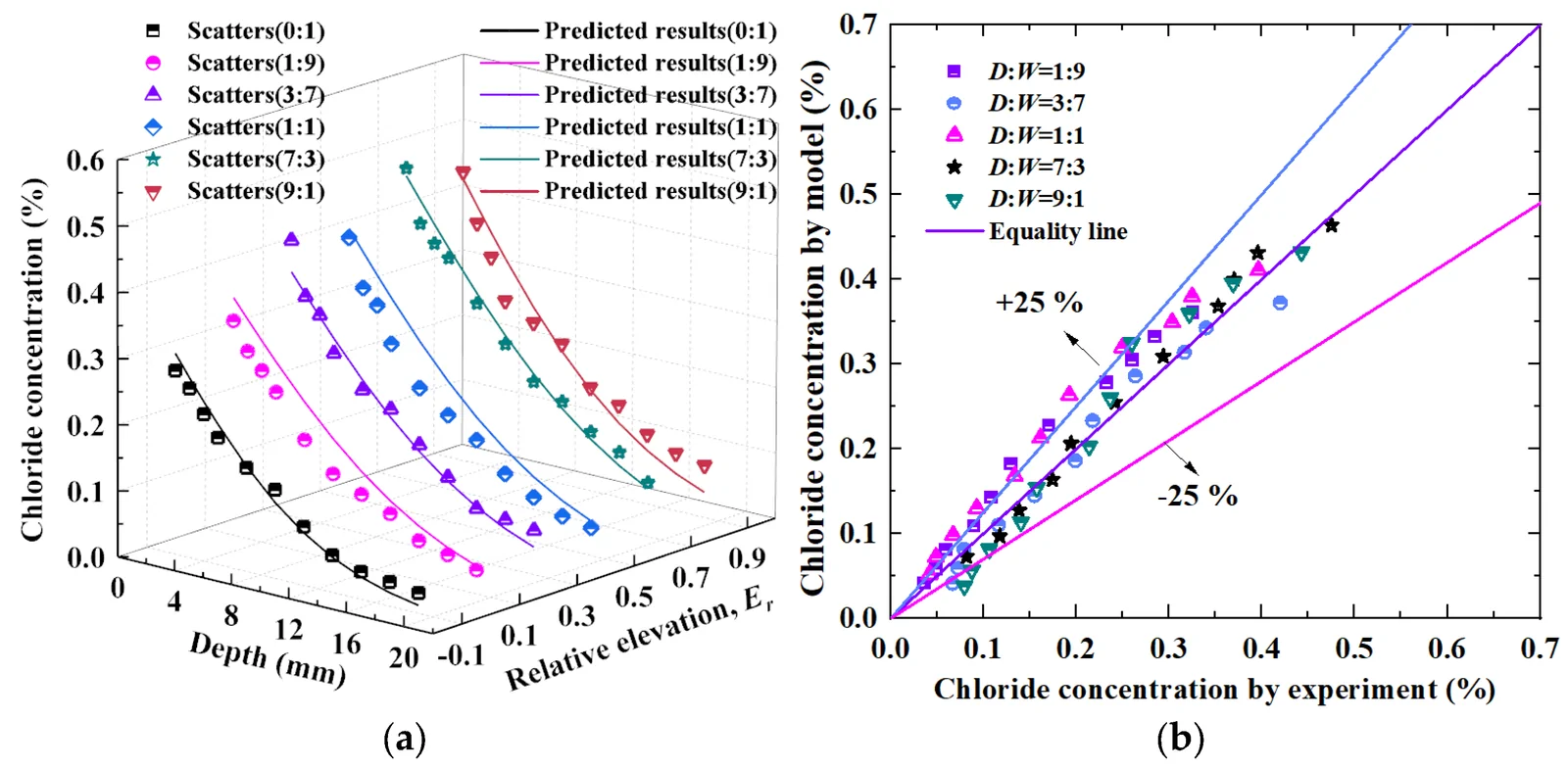
Comparison of the chloride concentrations estimated by the model in Equation (7) versus the experimental results when *t* = 100 d: (**a**) experiment vs. model; (**b**) quantitative comparison.

The above chloride transport prediction model, which considers the drying–wetting regime, relative elevation *E_r_*, exposure time t, and diffusion depth *x*, as shown in Equation (7), is essentially an empirical model based on multi-condition experimental data. Its development fully used the experimental data obtained at exposure durations of 30 d, 60 d, 150 d, and 200 d for regression analysis [29,30]. To quantitatively evaluate the applicability and predictive accuracy of the model under extreme saltwater intrusion and short-cycle wetting–drying alternation, this study selected the measured data at *t* = 100 d, which were independent of the fitting dataset, as the validation group. The measured concentration values were then compared with the model-calculated values. Figure 12 presents the validation comparison between the model predictions and the experimental data. The analysis indicates the following:

(1) Under different dry–wet ratios, namely different relative elevations, the depth-dependent attenuation trend of chloride concentration predicted by the model agreed closely with the measured scatter data. The model accurately captured the influence of short-cycle wetting–drying alternation on chloride concentration distributions in both the convection zone and the diffusion zone.
(2) The error analysis in Figure 12b shows that most relative errors between the model predictions and the measured concrete values were distributed within the ±25% confidence interval. This result confirms that the empirical model expressed by Equation (7) has good predictive performance and reliability. In summary, the model can effectively evaluate chloride concentration distributions at arbitrary depths and exposure times in concrete structures affected by different dry–wet ratios under extreme saltwater intrusion and short-term wetting–drying alternation. The model therefore provides a reliable tool for durability life prediction of concrete structures under similar complex service conditions.

Although the model showed satisfactory agreement within the tested range, its empirical nature imposes several limitations. The formulation assumes one-dimensional chloride ingress, a constant aging exponent m, and an empirical *N*(*E_r_*) relationship to represent elevation-dependent cyclic effects. Moreover, it was calibrated using one concrete mixture, uncracked specimens, a fixed 3.5% NaCl solution, and laboratory exposure cycles of 80 min and 24 h. Its present applicability is therefore limited to concrete and exposure conditions comparable to those investigated. For engineering use, the model can provide a relative-elevation-based framework for identifying chloride exposure severity and supporting durability assessment, but its parameters should be recalibrated using site and mixture-specific data. Future work will extend the experimental database to different water-to-binder ratios, supplementary cementitious materials, salinities, temperatures, cycle periods, cracked states, and field exposure conditions to evaluate and improve the model’s generalizability.

## 6. Conclusions

This study systematically investigated the chloride-induced deterioration characteristics of concrete exposed to the extreme high-salinity and short-term drying–wetting environment of estuarine ship locks through laboratory simulation tests and theoretical analysis, and the corresponding prediction model was developed. The main conclusions are as follows:

(1) The high-frequency short-cycle wetting–drying alternation caused by frequent filling and emptying operations in the ship lock significantly enhanced the convective transport efficiency in the concrete surface layer. The test results show that short-term dry–wet alternation induced strong concentration and crystallization accumulation of chloride ions in the surface convection zone, approximately within 2 mm of the surface, and the peak concentration was approximately 1.3 times that under a conventional long-term marine tidal environment.
(2) The surface chloride concentration and apparent chloride diffusion coefficient of concrete did not exhibit a simple linear increasing or decreasing relationship with the dry–wet ratio, expressed as relative elevation Er. Instead, both parameters showed pronounced nonmonotonic fluctuations, with an M-shaped or sawtooth-like distribution. The experimental data clearly show that the deterioration pattern first increased, then decreased, then increased again, and finally decreased, with a global extremum under the *D*:*W* = 7:3 condition (*E_r_* = 0.7). This behavior originated from the concentration-gradient driving force generated by the coupled action of capillary negative pressure formed during prolonged drying and strong adsorption during the subsequent short wetting stage. The capillary negative pressure produced by prolonged drying and the intense capillary adsorption induced by subsequent short wetting reached the most favorable synergistic coupling. Once the drying duration increased further, for example when the dry–wet ratio increased to 9:1, the excessively short wetting duration limited the effective infiltration carrier of the saline solution, causing the transport capacity to decrease. This finding confirms that the allocation of drying and wetting durations regulates chloride accumulation and diffusion in an extremely sensitive and nonlinear manner, which cannot be simply represented by a monotonic function.
(3) A bivariate chloride transport prediction model that simultaneously considers exposure time t and dry–wet ratio, represented by relative elevation Er, was established. By introducing the influence coefficient *N*(*E_r_*) for the drying–wetting regime and treating the aging attenuation exponent m as a constant, the model quantitatively evaluated the chloride concentration distribution in concrete structures subjected to short-cycle wetting–drying alternation under extreme saltwater intrusion. Most relative errors between the model predictions and measured values were controlled within ±25%, indicating that the model can accurately predict the chloride concentration distribution in the stable diffusion zone of concrete subjected to short-cycle wetting–drying alternation under extreme saltwater intrusion. The model provides a reliable theoretical basis for durability life assessment of concrete structures exposed to wetting–drying environments.

## Figures and Tables

**Table 1:** Mix proportion of mass concrete for the ship lock structure (unit: kg/m^3^) [14].

w/b	Cement	Fly Ash	GGBFS	Sand	Crushed Stone	Water	Water Reducer
0.38	189	130	72	788	1089	150	11.7

**Table 2:** Internal and external influencing factors for the concrete specimens.

No.	Internal/External Influencing Factor	Condition
1	Environmental chloride concentration	3.5%
2	Dry–wet ratio	0:1, 1:9, 3:7, 1:1, 7:3, 9:1
3	Drying–wetting alternation period	80 min, 24 h

## Data Availability

The original contributions presented in this study are included in the article. Further inquiries can be directed to the corresponding author.
